# In Vivo and In Vitro Starch Digestibility of Fresh Pasta Produced Using Semolina-Based or Wholemeal Semolina-Based Liquid Sourdough

**DOI:** 10.3390/foods10102507

**Published:** 2021-10-19

**Authors:** Simonetta Fois, Piero Pasqualino Piu, Manuela Sanna, Tonina Roggio, Pasquale Catzeddu

**Affiliations:** Porto Conte Ricerche Srl, Località Tramariglio, 07041 Alghero, Italy; fois@portocontericerche.it (S.F.); piupietro@gmail.com (P.P.P.); sanna@portocontericerche.it (M.S.); roggio@portocontericerche.it (T.R.)

**Keywords:** starch, available carbohydrates, glycemic response, glycemic index, glycemic load

## Abstract

The use of wholemeal flour and sourdough fermentation in different food matrices has received considerable attention in recent years due to its resulting health benefits. In this study, a semolina-based and a wholemeal semolina-based sourdough were prepared and added to the formulation of gnocchetti-type fresh pasta. Four types of gnocchetti were made, using semolina plus semolina-based sourdough (SS), semolina plus wholemeal semolina-based sourdough (SWS), semolina alone (S), and semolina plus wholemeal semolina (WS). The latter two were used as controls. The digestibility of starch was studied both in vitro and in vivo, and the glycemic response (GR) and glycemic load (GL) were determined. Starch digestibility, both in vivo and in vitro, was higher in wholemeal semolina than semolina pasta and the resulting GR values (mg dL^−1^ min^−1^) were also higher (2209 and 2277 for WS and SWS; 1584 and 1553 for S and SS, respectively). The use of sourdough significantly reduced the rapidly digestible starch (RDS) content and increased the inaccessible digestible starch (IDS) content. The addition of sourdough to the formulation had no effect on the GR values, but led to a reduction of the GL of the pasta. These are the first data on the GR and GL of fresh pasta made with sourdough.

## 1. Introduction

Pasta is a staple food, highly appreciated and consumed in several countries. In 2019 the world production of pasta reached almost 16 million tons and Italy was the leading pasta producer (3.5 million tons). Six Italians out of ten eat pasta on a daily basis, amounting to about 23.1 kg per capita per year of pasta consumed [1]. Dried pasta (less than 12.5% moisture content) represents the main global market share, while fresh pasta (more than 24.0% moisture content) represents a small but increasing share, particularly in the Italian market.

Pasta is commonly produced from durum wheat semolina but the addition of flours from other raw materials, such as rice, buckwheat, barley, spelt, millet, oats, quinoa, and legumes, has been investigated for the purpose of improving the nutritional quality and the health-promoting effects of pasta [2]. Many studies have investigated the effect of pasta fortified by a wide range of supplements, some of which, such as dietary fibers, amino acids, peptides and vitamins, can be considered functional ingredients that give pasta a potentially positive effect on health [3]. The consumption of fiber-rich pasta, or wholemeal pasta, has increased over the past few years, probably since it has well-recognized beneficial effects on human health, such as preventing cardiovascular disease, obesity, cancer, and diabetes risk factors [4,5]. Moreover, consumption of cereal fibers can increase the sensation of satiety and fullness [6], gut microbial diversity and abundance [7], and can also reduce the glycemic index, as observed in wholemeal bread by Scazzina et al. [8]. Unfortunately, decay of sensory and textural characteristics is observed when bran or wholemeal flour are incorporated into the foods [9], which reduces the demand for fiber-rich foods. To overcome these barriers, use of sourdough technology for wholemeal bread has been investigated in depth and has been reported to improve the sensory, nutritional and health quality of the bread itself. Fermented bran was successfully used to improve the sensory properties of bread containing bran [10]. An improvement in the sensory properties of fiber-rich pasta was observed when fermented wholemeal semolina was used [11].

Pasta is a good source of carbohydrates and the major dietary source of energy in the Mediterranean diet. It is considered a low glycemic index (GI) food [12], as glucose is liberated from carbohydrates fairly slowly after ingestion. The low GI of pasta is mainly due to its dense structure. This results in slow digestion and delayed gastric emptying, making pasta a unique example of a refined cereal food with a low GI. Pasta consumed in the context of a low-GI diet allows body weight to be reduced [13] and has a positive effect against diabetes risk factors [14] since it prevents the high postprandial glucose and insulin peaks, which may contribute to the development of insulin resistance and type 2 diabetes. In people with type-2 diabetes, consumption of pasta without exceeding the limits recommended for total carbohydrate intake, is not associated with a worsening of obesity or cardiovascular risk factors [12].

Sourdough fermentation has been shown to decrease the glycemic index in foods [8]. Use of sourdough technology has been investigated in fresh pasta made with fermented semolina and fermented wholemeal semolina [11,15], and data on the physical, chemical, and sensory properties of the pasta have been reported.

The aim of this work was to evaluate the effect of fermentation technology and the addition of wholemeal semolina, and the effect of their interaction on the in vitro digestion of carbohydrates and in vivo glycemic response of fresh pasta.

## 2. Materials and Methods

### 2.1. Raw Materials

Commercial wholemeal semolina (Integrale, Selezione Casillo S.r.l., Corato, Bari, Italy), and commercial semolina (Extra Arancio, Selezione Casillo S.r.l., Corato, Bari, Italy) were used. The percentage composition of the wholemeal semolina, as is or on a dry matter basis (D.M.), was: moisture 14.1%, ash 1.6% D.M., protein 12.5% D.M., fiber 7.8% D.M., dry gluten 8.5% D.M., gluten index 60%, alveographic W 199 (J × 10^−4^) and P to L ratio 5.12. The composition of semolina was: moisture 14.0%, ash 0.75% D.M., protein 13% D.M., fiber 2.7% D.M., dry gluten 11% D.M., gluten index 88%, alveographic W 176 (J × 10^−4^) and P to L ratio 1.31.

### 2.2. Preparation and Maintenance of Liquid Sourdough

The liquid sourdough starter was prepared according to Fois et al. [11] and was refreshed using a 5 L sourdough maker machine (Starpizza S.A.S., Verona, Italy). Two different sourdoughs were prepared: the first was a semolina-based sourdough prepared using water and semolina, while the second was a wholemeal semolina-based sourdough prepared with water and wholemeal semolina. Both were refreshed by daily back-slopping during which sourdough, water and semolina or wholemeal semolina were mixed in a ratio of 1:1:1, to obtain a dough yield of 200. The semolina-based sourdough had a pH value of 4.4 and a TTA of 10.0 mL NaOH (0.1 mol/L) in 10 g; the wholemeal-based sourdough had a pH value of 4.2 and a TTA of 13.0 mL NaOH (0.1 mol/L) in 10 g.

### 2.3. Fresh Pasta Making

Fresh pasta (of the gnocchetti sardi type) was prepared using the La Monferrina Dolly pasta maker (La Monferrina, Moncalieri, Italy) equipped with a bronze die. Four different pasta formulations were prepared:

Sample S: pasta made with semolina (1000 g) and water (300 g).

Sample SS: pasta made with semolina (700 g) and semolina-based sourdough (600 g). No extra water was added since the sourdough contained 50% of water.

Sample WS: pasta made with wholemeal semolina (300 g), semolina (700 g) and water (300 g).

Sample SWS: pasta made with whole meal semolina-based sourdough (600 g) and semolina (700 g). No extra water was added, as in sample SS.

After production, the fresh pasta was immediately pasteurized and packaged under modified atmosphere (CO_2_:N_2_ = 30:70), as in Fois et al. [15]. It was then stored at 4 °C until analysis.

### 2.4. Chemical Analysis of Cooked Pasta

The moisture content of the pasta was measured at 105 °C with a Thermogravimetric Analyzer Thermostep (Eltra GmbH, Haan, Germany). Total titratable acidity (TTA) and pH were determined with an automatic titrator (Crison, Hach Lange, Barcelona, Spain), after homogenizing a 10 g sample in 90 mL of distilled water. After 30 min of gentle stirring for sourdough and 60 min for chopped and homogenized pasta, the pH was determined and the samples were titrated to pH 8.5 with NaOH 0.1 mol/L. The TTA was expressed as mL of NaOH per 10 g of sample. Available carbohydrates (ACH) were determined (g/100 g of cooked pasta) using the Available Carbohydrates Assay Kit (Megazyme, Wicklow, Ireland). All the data have been reported in Table 1.

### 2.5. In Vitro Starch Digestibility

The pasta was cooked for the optimum cooking time and was then roughly chopped with a knife in order to simulate chewing. In vitro digestion was then performed as for Sanna et al. [16]. Digested samples were collected at 20, 60, 90, 120, and 180 min and the hydrolysis curves were built. Digestion was carried out in triplicate. The area under the hydrolysis curves (0–180 min) was calculated and the hydrolysis index (HI) was obtained as the ratio between the area under the curve of the sample and the area under the curve of the reference food (white breadcrumbs). The estimated glycemic index (GIe) was calculated following the Equation (1), as in Fico et al. [17]:

GIe = 8.198 + 0.862 × HI(1)

Rapidly digestible starch (RDS), slowly digestible starch (SDS) and inaccessible digestible starch (IDS) were calculated. RDS is the glucose released after 20 min of in vitro digestion. SDS and IDS are defined as the glucose released within the 20 and 120 min time-frame and within the 120 and 180 min time-frame, respectively. IDS is defined as “inaccessible digestible starch” since it is not actually digestion-resistant starch but just physically inaccessible to the digestive enzymes. It was made accessible by homogenizing the sample after 120 min of in vitro digestion [16].

### 2.6. Glycemic Response (GR) and Glycemic Load (GL)

Glycemic response was determined in vivo following the procedures reported by the Food and Agriculture Organization/World Health Organization [18] and the method reported by Sugiyama et al. [19]. The study was approved by the Sardinian Ethical Committee of the Azienda Tutela Salute (ATS). Fifteen volunteers (seven female and eight male) were recruited by the Porto Conte Ricerche laboratory (Alghero, Italy), where the study was conducted. Volunteers aged between 25 and 55 years old were chosen on the basis of their body mass index (BMI), which had to range between 18.5 and 24.9 kg m^−2^. BMI was calculated as the ratio of weight (kg) to squared height (m), as indicated in: http://www.salute.gov.it/portale/salute/p1_5.jsp?id=135&area=Vivi_sano (accessed on 21 December 2020).

The volunteers were informed about the main criteria required in order to be eligible to take part in the study (i.e., they had to be healthy, not affected by chronic diseases, free from food intolerances and allergies, not involved in drug usage, not in a special physiological condition, such as breastfeeding) and what they would be asked to do. The volunteers were then asked to formalize their membership by signing an agreement. Obviously, each one was free to withdraw from the experimentation at any time without explanation.

#### 2.6.1. Experimental Procedures

The pasta was cooked for its optimum cooking time, according to the AACC Approved Method 66-50 [20], in previously salted (1.3% *w*/*v*) boiling water. The ratio of pasta to cooking water was 25 g per 300 mL. The pasta was served as is, with no seasoning. The four types of pasta were served to the volunteers on different days, in a randomized order so that none of the volunteers exceeded one meal per two weeks, and were tested for equivalent available carbohydrate content (50 g). Monohydrate glucose solution (Glucose (Monico) 50% p/V, Monico SPA, Venezia, Italy) was used as reference food and served twice to the volunteers. The test was conducted at around 09:00 a.m. The volunteers were given the following instructions: prior to the test they had to fast for 12 h during which they were allowed to drink water *ad libitum*. On the evening before the test, they were allowed to have dinner as usual with the exception of alcohol, which was forbidden. On the test day, blood glucose after fasting was measured at time zero (T0), after which the volunteers consumed pasta or the reference food within a period of 15 min and were given 250 mL of water. Blood glucose was then measured at 15, 30, 45, 60, 90, and 120 min after the volunteers had started to eat. The volunteers were asked to minimize their physical activity during the test. Capillary blood samples were obtained by the finger prick method (Glucoject Dual Plus, A.Menarini diagnostics S.r.l., Firenze, Italy) and glucose was measured using a Glucomen Lx3 calibrated self-monitoring system (A. Menarini diagnostics S.r.l., Firenze, Italy) with a published <3.6% analytical coefficient of variation (CV).

#### 2.6.2. Data Manipulation

The curves of blood glucose increase (µmol ml^−1^) versus time (from 0 to 120 min) were built for each volunteer after ingestion of the glucose solution and pasta samples (S, WS, SS and SWS). Blood glucose increases were calculated by subtracting the blood glucose concentration value at T0 from the blood glucose concentration values measured at any following time point. In this way, the blood glucose value at T0 was zero for all the curves. The total glucose response (GR) was calculated for each curve as the total area under the curve, by adding the incremental areas under the curve (IAUC) approximated with the trapezoid rule for time frames 0–15, 15–30, 30–45, 45–60, 60–90, and 90–120 min. The height of the rectangular trapezium was the time difference on the x-axis between two subsequent time points (min), whereas the major and minor basis were the values of the blood glucose increases on the y-axis (µmol ml^−1^), corresponding to the two time points. Finally, the percentage ratio between the GR of the pasta and the GR of the glucose solution, used as reference food, was calculated and hereafter referred to as “apparent glycemic index” (GI_a_). The glycemic load (GL) was then calculated using the following Equation (2):

GL = (GI_a_ × grams of carbohydrate in the standard serving size/100) (2)

### 2.7. Statistical Analysis

The standard ANOVA procedure (randomized complete design with 2^2^ treatments) was applied to the dataset. The experiment involved two factors: with and without the addition of sourdough, and with and without the addition of wholemeal semolina. A multiple comparison procedure was applied for the GR values to determine if the mean values of each sample were significantly different from that of the reference food. The mean values were separated by LSD test at *p* = 0.05 significance level, using the Statgraphics Centurion 18 software package (version 18, Statpont Technologies Inc., Warrenton, VA, USA).

## 3. Results and Discussion

### 3.1. In Vitro Starch Digestion

**Figure 1:**
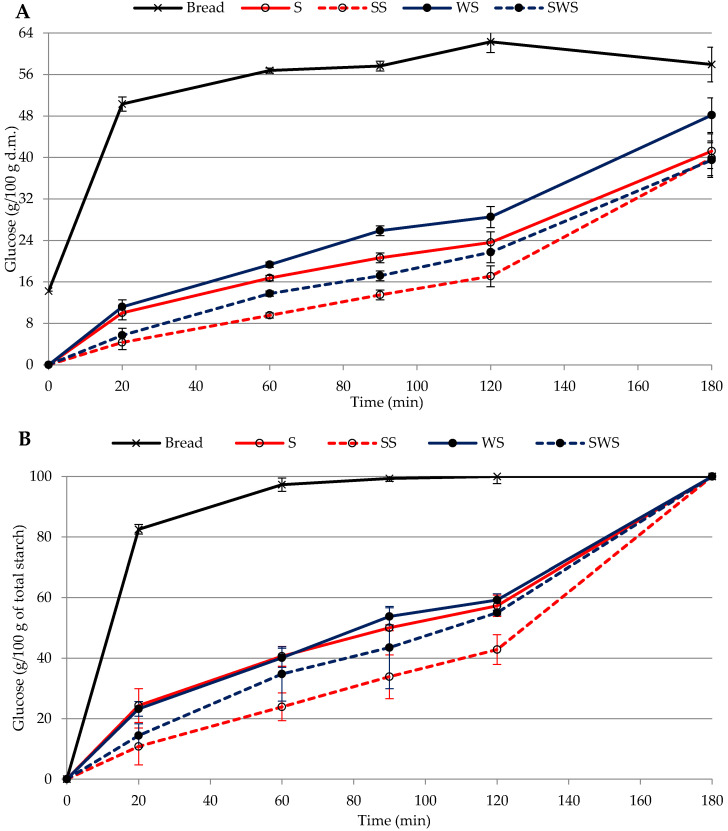
Glucose values from in vitro starch digestion of pasta samples. (**A**) Glucose with respect to dry pasta or bread (g/100 g). (**B**) Glucose with respect to total starch (g/100 g). S, pasta with semolina. SS, pasta with semolina-based sourdough. WS, pasta with wholemeal semolina. SWS, pasta with wholemeal semolina-based sourdough. Bars indicate LSD intervals at 95% confidence level.

The amount of glucose released from starch during in vitro digestion of the four samples of pasta is reported in Figure 1A,B, while the GIe data are reported in Table 2. The shape of the curves obtained was similar for the four types of pasta and, as expected, the glucose values were far lower than the values obtained from the in vitro digestion of white breadcrumbs reported in the same figure.

In vitro starch hydrolysis of both pasta samples with wholemeal semolina (WS and SWS) released more glucose than the corresponding samples prepared with semolina, indicating that the presence of fiber increased starch availability to the hydrolytic enzymes, in pasta both with and without sourdough. As a consequence, the values of GIe were significantly higher in WS (38.5) than in S (33.2) and in SWS (28.6) than in SS (23.6). This result is in accordance with Vignola et al. [21], who found a higher amount of hydrolyzed starch in wholemeal pasta and postulated that fiber may disrupt protein matrix and give rise to a porous structure that facilitates the action of hydrolytic enzymes. On the contrary, other researchers reported a reduction in starch digestion after the addition of fiber in the pasta formulation. Padalino et al. [22] observed lower starch hydrolysis in wholemeal spaghetti than in semolina spaghetti. A reduction in starch hydrolysis was also observed in pasta made using quinoa flour compared to semolina pasta [23] and the reason was suggested to be due to the high concentration of dietary fiber in quinoa flour. The data reported in Figure 1A show that use of sourdough had a significant effect on the hydrolysis of starch. The hydrolysis curves of pasta with sourdough (SS and SWS) showed lower values of released glucose at any time the analyses were performed, while the GIe was significantly lower than the corresponding pasta made without sourdough (S and WS). This was in accordance with the results obtained by Lorusso et al. [23], who observed a significant decrease in starch hydrolysis in pasta made with fermented quinoa. The lowest values of hydrolyzed starch were found in pasta containing semolina-based sourdough (SS) (Figure 1A). Similar results were observed in sourdough bread, where the degree of in vitro starch digestion was found to be lower than in yeasted bread, with a further decrease in sourdough bread enriched with fibers [24,25], contrary to this work. Reduced starch hydrolysis in pasta with sourdough can be explained as an effect of biological acidification, which creates interactions between gluten and starch, and hinders the access of enzymes to the starch [26].

When the data are expressed as percentage of starch digested over the total (Figure 1B), it is worth mentioning that the rate of hydrolysis in pasta with semolina and pasta with wholemeal semolina is similar. This in agreement with Bustos et al. [27] who reported that the kinetic constant during wholemeal pasta digestion did not differ from that observed in white wheat-based pasta, although starch hydrolysis was higher in wholemeal pasta, as in our data. In the same way, the effect of fermentation is still evident when the data are expressed as percentage of starch digested over the total.

The values of total starch (TS) at 180 min, rapidly digestible starch (RDS), slowly digestible starch (SDS) and inaccessible digestible starch (IDS) were calculated as percentages of TS and are reported in Table 2. Both use of sourdough and use of wholemeal semolina had a significant effect on the values of the starch fractions (Table 2).

The highest value of TS was found in WS pasta (48.1%). It was similar to the value found in white bread and was higher than that of S pasta (41.2%) The value decreased in SWS pasta (39.5%) as an effect of the use of sourdough. Use of sourdough only had an effect on TS in the presence of wholemeal semolina, whereas no differences were detected between S and SS pasta. Demirkesen-Bicak et al. [28] found a lower value of TS in wholemeal bread than in white bread, both in the one fermented with baker’s yeast and sourdough, while sourdough fermentation had no effect on TS. As highlighted by Bustos et al. [27], the disintegration kinetics of food directly affects the level of starch hydrolysis, as the more the structure of the food is porous the higher the starch hydrolysis level will be, while the whole wheat bread described by Demirkesen-Bicak et al. [28] had a more compact structure with a lower specific volume than white bread. On the contrary, wholemeal pasta has a more porous structure than semolina pasta due to the presence of insoluble bran fiber, which may disrupt the protein matrix, also leading to a weakening of the structure [27]. The more compact structure of semolina pasta results in a very close protein network, which entraps starch granules and delays α-amylase activity [21]. It has been demonstrated that the presence of bran from wholemeal semolina weakens the pasta structure [29] since it probably interferes with the formation of such a continuous matrix. This hypothesis also explains why the SDS value reported in Table 2 rose from 33.0% in S to 35.9% in WS and from 30.9% in SS to 40.6% in SWS, while the IDS value decreased when wholemeal semolina was used (from 45.6% in S to 40.8% in WS and from 56.7% in SS to 45% in SWS). In fact, it is worth mentioning that IDS is defined as inaccessible digestible starch, which is starch resistant to enzymatic digestion because of the food structure [30]. IDS should include both type 1 and type 3 resistant starch (RS), the former being potentially digestible starch, but physically inaccessible to hydrolytic enzymes, and the second being retrograded starch from food processing [31]. In our case, starch was digested immediately after the thermal treatment of cooking, when the starch was fully gelatinized, so we can infer that almost all the IDS is represented by type 1 resistant starch [16].

The use of sourdough had a significant and positive effect on the IDS content of pasta. In actual fact, an increase in IDS was found when sourdough was used, in both semolina and wholemeal semolina pasta. There is a lack of literature on the use of sourdough in fresh pasta, but data reported by Fois et al. [15] are in line with these data. Demirkesen-Bicak et al. [28] confirmed the increase in resistant starch as an effect of sourdough fermentation, although these data refer to bread, in which case the resistant starch comprises both types 1 and 3.

An explanation of the higher level of IDS in pasta with sourdough can be inferred from the observation of Ӧstman et al. [26], who suggested that the organic acids present in sourdough bread during thermal treatment can promote the formation of starch-gluten interactions, which make the food structure less susceptible to hydrolytic enzymes in the first two hours of digestion. Starch-gluten interactions contribute to type 1 resistant starch. This is confirmed by the lower amount of RDS in pasta with sourdough as compared to pasta without sourdough, in both the semolina and wholemeal semolina samples. Use of sourdough reduced the RDS and increased the IDS levels. These results could have an important implication from a nutritional point of view, as the target outcomes in food processing focus on lowering the RDS and raising the RS content.

### 3.2. Glycemic Response and Glycemic Load

**Figure 2:**
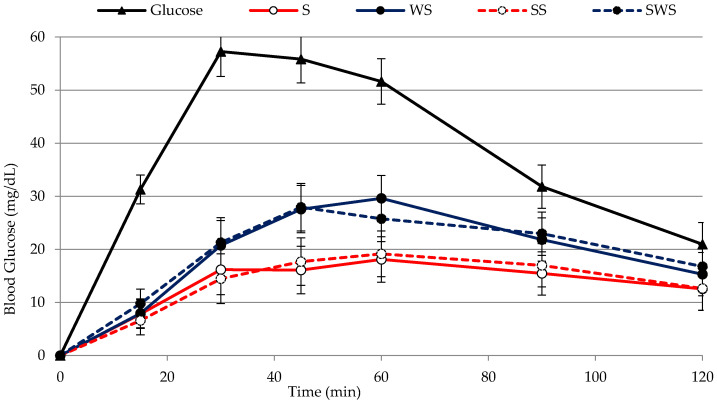
Blood glucose values obtained after in vivo digestion of the four pasta samples. S, pasta with semolina. WS, pasta with wholemeal semolina. SS, pasta with semolina-based sourdough. SWS, pasta with wholemeal semolina-based sourdough. Bars indicate LSD intervals at 95% confidence level.

The curves of glucose increases versus time and the GR values are reported in Figure 2 and Table 2, respectively, for the four pasta samples and for the glucose solution used as a reference food.

A comparison of the differences between the mean GR value of each of the pasta samples and the mean GR value of the reference food, showed that such differences were, as expected, highly significant (*p* < 0.001). The data in Table 2 show that use of wholemeal semolina had a significant effect (*p* < 0.001) on glycemic response. On the contrary, sourdough had no effect on glycemic response (Table 2) and the curves corresponding to S and SS, and to WS and SWS were very close (Figure 2). GI_a_ was calculated as the percentage ratio between the incremental area obtained after ingestion of pasta and the incremental area obtained after ingestion of the reference food, both containing 50 g of available carbohydrates (ACH). The GI_a_ values were 38.0 and 41.0 for S and SS pasta, and 57.0 and 55.5 for WS and SWS pasta, respectively. Statistical analysis revealed that use of sourdough had no effect on GI_a_, whereas use of wholemeal semolina did (Table 2), and that there was no interaction between the two factors (wholemeal semolina and sourdough). Foods are commonly divided into three classes on the basis of their GI, i.e., foods with low GI (<55); foods with intermediate GI (55–70); foods with high GI (>70). According to this classification, the values obtained here indicate that the addition of wholemeal semolina to the pasta formulation raised the GI_a_ of the pasta from low to medium.

The effect of the use of wholemeal semolina was unexpected. Henry et al. [32] reported that there was no difference between the GI values of Fusilli pasta and whole wheat Fusilli pasta. Atkinson et al. [33] reported that the average GI of white spaghetti (49) and wholemeal spaghetti (48), derived from multiple studies by different laboratories, was the same. Kristensen et al. [34] reported that fiber had no effect on postprandial glycemia, when comparing refined wheat pasta and whole wheat pasta-based meals. Those authors hypothesized that this lack of fiber having an effect might be related to the type of dietary fiber present in wheat which, as it does not form a viscous solution upon hydration in the gastrointestinal tract, probably does not delay gastric emptying. In this work, the higher glycemic response of wholemeal-based pasta observed in vivo was in agreement with the data obtained after in vitro starch hydrolysis (Figure 1), which suggests that the fiber favored the accessibility of hydrolytic enzymes to starch granules, both in vitro and in vivo. It should be noted that no data can be found on the glycemic response of a pasta such as the one analyzed in this work (i.e., fresh and pasteurized gnocchetti-type pasta), and that all available data in literature are on dry pasta, mainly the spaghetti type. Scazzina et al. [35] published the GI of certain commercial Italian foods, among which pasta, showing how the GI of wholemeal spaghetti can range from low (35) to almost medium (55) depending on the brand. Meaning that different processing conditions can drastically modify the GI value.

In this study, use of sourdough had no effect on the glycemic response of fresh pasta, contrary to what was found in literature for bread, which reported how sourdough fermentation or the addition of organic acids had been used to lower the glycemic index [36]. This, as organic acids were thought to delay gastric emptying (mainly acetic acid), or to promote starch-gluten interactions which reduce starch bioavailability (mainly lactic acid) after heat treatment [26]. Scazzina et al. [37] found, for bread, that neither the leavening technique nor fiber content influenced starch availability to hydrolytic enzymes in vitro, and that the leavening technique significantly affected glucose response in vivo, whereas fiber content did not, suggesting that organic acids could delay gastric emptying without influencing starch availability. A possible explanation of our in vivo data cannot be related to the loss of organic acids in the cooking water, as reported by Fois et al. [11], although the pasta was cooked in water before in vitro digestion, where an effect of fermentation was detected. The pasta for in vivo measurements was cooked in salted water to make it palatable, whereas the pasta for in vitro measurement was not. A possible effect of salt on the gluten network and on starch gelatinization might have interfered with the effect of sourdough fermentation and needs further investigation.

It is worth mentioning that the increase in postprandial glycemia is not only related to the GI of a food but also to the amount of carbohydrates in the serving size of that food. Thus, another index, glycemic load (GL), was used [36]. In this study GL was calculated as the product of GI_a_ and the grams of available carbohydrate in 160 g of cooked pasta (standard serving size) divided by 100. As reported in Table 1, the amount of ACH in cooked semolina-based pasta (39.24% in S and 33.28% in SS) was higher (*p* < 0.05) than that of wholemeal-based pasta (34.27% in WS and 31.32% in SWS). Then, the serving size of pasta containing 50 g of ACH consumed by the volunteers, was different (i.e., 127 g of S and 150 g of SS were weighed), whereas in the case of WS and SWS the weight was 146 and 159 g, respectively. This result was in accordance with a study by Henry et al. [32], who reported a lower content of available carbohydrates and, therefore, a larger serving size for whole wheat Fusilli than semolina Fusilli. Moreover, even sourdough wholemeal bread was found to have a lower content of available carbohydrates than yeast leavened bread [38]. The GL values were 23.9 for S, 21.8 for SS, 31.3 for WS, and 27.8 for SWS. The effect of using wholemeal semolina was still evident in GL, but a new effect of the use of sourdough, which reduced the GL value, could be detected giving pasta with sourdough an increased value from a nutritional point of view.

## 4. Conclusions

Pasta is a popular carbohydrate-based food with a low glycemic index, which can be fortified with a variety of ingredients to improve its nutritional qualities. In this paper, the digestibility of starch was studied, in vitro and in vivo, in pasta with the addition of wholemeal semolina and sourdough. The results showed that use of wholemeal semolina made the gluten matrix more susceptible to the in vitro activity of hydrolytic enzymes. This result was confirmed by glucose determination after in vivo digestion; unexpectedly the GI_a_ value of wholemeal pasta was higher than that of semolina pasta. However, the data available in literature concern dry pasta, mostly the spaghetti type, whereas gnocchetti-type fresh and pasteurized pasta was prepared for this study. It is known that food processing conditions drastically affect the activity of hydrolytic enzymes and, as a consequence, the GI of foods. Use of sourdough had no effect on the GR value, whereas the GL value decreased owing to the effect of the reduced content of available carbohydrates in pasta made with sourdough. It is rather difficult to compare these results with the data available in literature, which is focused on the use of sourdough in bread making. The physical characteristics of the matrices are deeply different. In the case of bread, the crumb is a porous matrix with a high surface to volume ratio. It is therefore more susceptive to the attack of hydrolytic enzymes than pasta which, as a consequence of the extrusion process, has a more compact structure and a reduced surface to volume ratio. Moreover, pasta is cooked in an excess of hot water, in which some of the starch is leached. Finally, pasta is eaten immediately after cooking, without leaving time for the retrogradation phenomenon to fully take place.

## Figures and Tables

**Table 1:** Chemical properties of cooked pasta.

Pasta	Moisture (g/100 g)	pH	TTA ^1^ (mL NaOH N/10)	Available Carbohydrates ^2^
S	57.48 ± 0.59	6.53 ± 0.07	0.85 ± 0.03	39.24 ± 1.34
SS	56.76 ± 0.44	5.52 ± 0.17	2.01 ± 0.44	33.28 ± 1.69
WS	58.32 ± 2.24	6.61 ± 0.02	1.24 ± 0.06	34.27 ± 0.82
SWS	55.85 ± 1.13	5.79 ± 0.04	2.32 ± 0.01	31.32 ± 0.58

(S, pasta with semolina; SS, pasta with semolina-based sourdough; WS, pasta with wholemeal semolina; SWS, pasta with wholemeal semolina-based sourdough. Mean value of at least four replicates ± standard deviation. ^1^ TTA, total titratable acidity expressed as mL of 0.1 N NaOH/10 g of pasta dry matter. ^2^ Grams of glucose in 100 g of cooked pasta, as is basis).

**Table 2:** Results of in vivo digestion and in vitro starch hydrolysis of pasta.

	GR	GI_a_	GIe	GL	TS	RDS	SDS	IDS
Samples	Mg dL^−1^ min^−1^				g/100 g Pasta	g/100 g TS		
S	1584 ^b^	38.0 ^b^	33.2 ^b^	23.9 ^c^	41.2 ^b^	24.8 ^a^	33.0 ^b^	45.6 ^b^
SS	1553 ^b^	41.0 ^b^	23.6 ^d^	21.8 ^d^	39.9 ^b^	10.7 ^b^	30.9 ^b^	56.7 ^a^
WS	2209 ^a^	57.0 ^a^	38.5 ^a^	31.3 ^a^	48.1 ^a^	23.2 ^a^	35.9 ^a^	40.8 ^c^
SWS	2277 ^a^	55.5 ^a^	28.6 ^c^	27.8 ^b^	39.5 ^b^	14.4 ^b^	40.6 ^a^	45.0 ^b^
Reference Food	4484	100	100		48.1	82.5	27.4	0
					Significance			
Sourdough	ns	ns	***	*	*	**	ns	*
WholemealSemolina	***	***	***	*	*	ns	*	*
Sourdough*WholemealSemolina	ns	ns	ns	ns	***	ns	ns	ns

*** *p* < 0.001; ** *p* < 0.01; * *p* < 0.05; ns, not significant. The different superscript letters in the column denote a statistically significant difference at *p* ≤ 0.05. GR, glucose response. GI_a_, apparent glycemic index. GIe, glycemic index estimated after in vitro digestion. GL, glycemic load per 160 g serving size. TS, total starch. RDS, rapidly digestible starch. SDS, slowly digestible starch. IDS, inaccessible digestible starch.

## Data Availability

The data presented in this study are available on request from the corresponding author.
